# Efficacy of lofexidine for mitigating opioid withdrawal symptoms: results from two randomized, placebo-controlled trials

**DOI:** 10.1080/21556660.2019.1704416

**Published:** 2020-01-08

**Authors:** Danesh Alam, Carlos Tirado, Mark Pirner, Thomas Clinch

**Affiliations:** a Northwestern Medicine Central DuPage Hospital, Winfield, IL, USA; b CARMA Health, PLLC, Austin, TX, USA; c US WorldMeds, LLC, Louisville, KY, USA

**Keywords:** Opioid withdrawal, opioid withdrawal syndrome, opioid use disorder, lofexidine, alpha_2_-adrenergic agonist, addiction, detoxification, opioid dependence

## Abstract

**Objectives:**

Fear of opioid withdrawal syndrome (OWS) often dissuades opioid discontinuation. Lofexidine is an FDA-approved, alpha_2_-adrenergic receptor agonist for treatment of OWS. Pivotal trial results from the per-protocol statistical analyses have been published. However, the FDA prescribing information presents these efficacy results using a different, standardized statistical approach that does not transform data or impute missing values. This analysis is easier to interpret and allows comparison across studies. This reanalysis is presented here.

**Methods:**

Studies were double-blind, placebo-controlled for 7 days in Study 1 and 5 days in Study 2. Opioid-dependent adults received placebo or lofexidine; efficacy was assessed using the Short Opioid Withdrawal Scale of Gossop (SOWS-G) daily.

**Results:**

Study 1 (*N* = 602) mean SOWS-G scores were 6.1 (SE: 0.35), 6.5 (SE: 0.34), and 8.8 (SE: 0.47) over Days 1–7 for lofexidine 2.88 mg/day, 2.16 mg/day, and placebo, respectively (for 2.88, *p* < .0001; for 2.16 mg, *p* < .0001). Study 2 (*N* = 264) mean SOWS-G scores were 7.0 (SE: 0.44) and 8.9 (SE: 0.48) over Days 1–5 for lofexidine 2.16 mg/day and placebo, respectively (*p* = .0037). Median time to treatment discontinuation was approximately 2 days later with lofexidine treatment than with placebo and significantly more lofexidine-treated subjects completed the studies. Hypotension and bradycardia were more common with lofexidine. More placebo subjects withdrew prematurely for lack of efficacy.

**Conclusion:**

This simplified analysis confirmed previous per-protocol results, that lofexidine better reduces OWS severity and increases retention compared with placebo in opioid-dependent adults. These results are robust and comparable across studies using various methods of analysis.

**ClinicalTrials.gov identifier:**

Study 1, NCT01863186; Study 2 NCT00235729. URL: https://clinicaltrials.gov/

## 1. Introduction

Opioid withdrawal syndrome (OWS) is a significant, incapacitating complication of abrupt opioid discontinuation in opioid-dependent individuals. Symptoms commonly include anxiety, insomnia, tremors, pain, muscle spasms, and GI upset^1^^,^^2^. Distress caused by OWS is especially severe during the first several days after withdrawal of short-acting opioids^3^^,^^4^ and fear of OWS is a substantial barrier to opioid discontinuation^5^^,^^6^.

A major physiologic driver of OWS is central noradrenergic hyperactivity that results when opioids are abruptly discontinued in opioid-tolerant individuals^7^. Lofexidine is a non-opioid medication that acts as an agonist at central alpha_2_-adrenergic presynaptic receptors and thereby suppresses noradrenergic hyperactivity^8^. Lofexidine has been approved in the UK for OWS since the 1990s and was approved by the FDA in May of 2018 for mitigation of opioid withdrawal symptoms to facilitate abrupt opioid discontinuation in adults.

Pivotal study data analyses presented in the lofexidine prescribing information (label; LUCEMYRA, US WorldMeds LLC) differ from the previously published study reports^9^^,^^10^. Specifically, the main efficacy endpoint (Short Opioid Withdrawal Scale of Gossop [SOWS-G]^2^ score change) and subject retention rates were analyzed and presented to standardize analyses across the two studies and improve ease of data interpretation. The label analyses differed in that observed, non-transformed data were used to avoid log-transformed scores and imputation of missing data values. The purpose of this paper is to present the statistical methodology and results presented in the FDA prescribing information and to compare results across studies.

## 2. Materials and methods

For more detailed methods, see the previously published reports^9^^,^^10^.

### 2.1. Overview of study designs

Both trials were randomized, double-blind, placebo-controlled, inpatient studies conducted at multiple sites in the United States. The ClinicalTrials.gov identifier was NCT01863186 for Study 1 and NCT00235729 for Study 2. Both protocols were approved by central or local institutional review boards at all study sites. Written, informed consent was obtained from all subjects prior to performing any study procedures.

**Figure 1.**
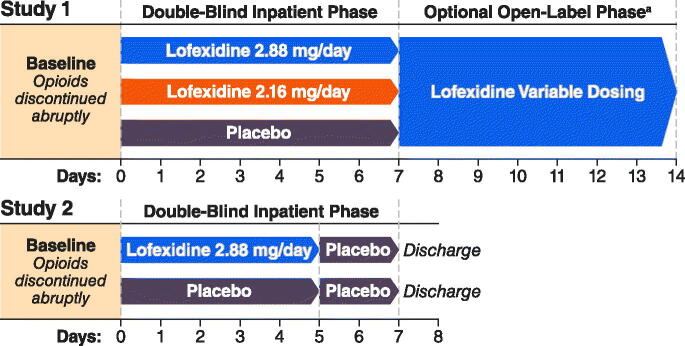
Trial designs. ^a^Note: Results from the open-label phase of Study 1 are not presented.

Study 1 enrolled subjects from June 2013 to December 2014. Study 2 enrolled subjects from June 2006 to October 2007. Figure 1 depicts the trial designs. Study 1 evaluated lofexidine doses 2.16 mg/day (0.54 mg QID), 2.88 mg/day (0.72 mg QID) compared with placebo (randomized 3:3:2) for 7 days. Study 2 evaluated lofexidine 2.88 mg/day (0.72 mg QID) compared with placebo (randomized 1:1) for 5 days and all subjects received placebo on study days 6 and 7.

In both studies, a baseline score of ≥2 on the Objective Opiate Withdrawal Scale on the morning prior to randomization was required to confirm that subjects were entering opioid withdrawal.

### 2.2. Participants

Adults (≥18 years old) with dependence on short-acting opioids and self-reported use on ≥21 of the past 30 days who voluntarily consented to enter the study were enrolled. Opioid-dependence was determined using the Mini International Neuropsychiatric Interview in Study 1 and the Structured Clinical Interview Axis I in Study 2. Major exclusion criteria included use of methadone or buprenorphine in the past two weeks, unstable/serious medical or psychiatric illness, pregnancy or lactation, self-reported positive HIV status, and use of psychotropics, antihypertensives, antiarrhythmics or anticonvulsants within the past four weeks. An abnormal cardiovascular exam, including prolonged corrected QT interval (>450 ms for males, >470 ms for females) and significant hypertension or hypotension was cause for exclusion.

### 2.3. Study drug dosing

Study drug was dosed QID in both studies: lofexidine 2.88 mg/day (0.72 mg QID), lofexidine 2.16 mg/day (0.54 mg QID) and placebo in Study 1 (randomized 3:3:2) and lofexidine 2.88 mg/day (0.72 mg QID) and placebo in Study 2 (randomized 1:1). Lofexidine or placebo was dosed at 8 am, 1 pm, 6 pm, and 11 pm. The following supportive medications were allowed by protocol: guaifenesin, antacids, dioctyl sodium sulfosuccinate, psyllium hydrocolloid suspension, bismuth sulfate, acetaminophen, and zolpidem. Any additional medications required approval by the Sponsor’s medical monitor before administration.

### 2.4. Randomization and blinding

Both studies used randomization procedures that took gender into account when allocating treatment assignment to assure adequate lofexidine exposure was achieved in females. In Study 1, a stratified randomization procedure separately allocated males and females to one of the three treatment groups in a 3:3:2 ratio (lofexidine 2.16 mg, lofexidine 2.88 mg, or placebo). In Study 2, a “biased coin” procedure was used which allocated subjects in a 1:1 ratio (lofexidine 2.88 mg or placebo) using randomization probabilities favoring the treatment group with a “deficit” in enrollment based on the subject’s gender^11^. Lofexidine or matched placebo was provided in blister cards. All study personnel, the sponsor and study subjects were blinded to treatment assignment.

### 2.5. Endpoints

#### 2.5.1. Primary efficacy

OWS severity as measured by the SOWS-G^2^ scale was the efficacy outcome measure for both studies. SOWS-G is a 10-item, patient-reported outcome with a total score ranging from 0 to 30 whereby lower scores indicate less severe opioid withdrawal. The SOWS-G items include: feeling sick, stomach cramps, muscle spasms/twitching, feeling of coldness, heart pounding, muscular tension, aches and pains, yawning, runny eyes, and insomnia/problems sleeping. In both studies, SOWS-G was measured at baseline and daily 3.5 h after the first morning dose (8:00 am) of study drug. Changes of 2–4 points in SOWS-G scores have been correlated to a clinically meaningful response^12^.

#### 2.5.2. Other efficacy

The other key efficacy variable was study completion rate during the double-blind treatment period, assessed as 7-day completers in Study 1 and 5-day completers in Study 2. Kaplan-Meier retention analyses are also presented for both studies. Other secondary efficacy endpoints are not presented but have been previously published^9^^,^^10^.

#### 2.5.3. Safety

Treatment-emergent adverse events (AEs) were collected on a daily basis. Vital signs were closely monitored pre- and post-dosing throughout both studies. ECGs were acquired at baseline and on Days 1, 2, 4, and 7 in Study 1 and daily in Study 2. In Study 1 hypotension and bradycardia were to be reported as adverse events based on predefined limits (systolic blood pressure <90mm Hg, diastolic blood pressure <50mm Hg, pulse rate <50 beats/min, or >20% decrease from screening; decrease in standing systolic or diastolic blood pressure >25% from recumbent values) independent of whether symptoms were present.

### 2.6. Sample size calculations

The sample size calculation for Study 1 was based on results from Study 2 (which was completed several years earlier). A random coefficients model was used to estimate treatment effect and subject variability with respect to SOWS-G scores. The treatment effect for lofexidine 2.88 mg versus placebo was estimated based on area under the curve for SOWS-G scores over Days 1–7. With a sample size of 600, the power to detect a significant difference between lofexidine 2.88 mg and placebo was 95%.

The sample size for Study 2 was based on results from an even earlier lofexidine trial^13^. The calculation assumed a 1:1 randomization of subjects to placebo or lofexidine, a 35% discontinuation rate, a minimal clinically significant difference on SOWS-G of five points, and a standard deviation of 10. With a sample size of 264, the power to detect a significant difference between lofexidine 2.88 mg and placebo was 90%.

### 2.7. Statistical analyses of efficacy data

A statistical methodology from the original per-protocol analyses is described below to illustrate the differences from the label analysis. The previous results have been presented and published elsewhere and are not included in this report^9^^,^^10^.

#### 2.7.1. Per-protocol analyses

Per-protocol primary endpoints were mean log-transformed SOWS-G score over Days 1–7 for Study 1 and mean SOWS-G score on Day 3 for Study 2. For the Study 1 per-protocol analysis, a pattern mixture model was used; lofexidine subject missing data were imputed with placebo values as the most conservative approach. For the Study 2 per-protocol analysis, an analysis of covariance model was used; missing data were imputed from completer data derived from the same treatment group. For Study 1, all randomized subjects who received study drug were included in the SOWS-G analysis. For Study 2, subjects who received study medication and had at least 1 post-medication SOWS-G score were analyzed for the primary SOWS-G analysis.

Study 1 study completion rate was analyzed using a logistic regression model including fixed effects for treatment group and sex and was calculated for all subjects who were randomized and treated. Study completion was defined as having taken at least 1 dose of study medication on Day 7 and completed the post-dose SOWS-G assessment on Day 7. Study 2 completion rate was analyzed using Fisher’s Exact test and was calculated for all randomized subjects. Study completion (5-day treatment) was defined as having completed the 5-day treatment phase and discharged on Day 6 or later.

#### 2.7.2. Label analyses

In order to standardize results across studies, efficacy endpoints were analyzed during the double-blind periods, Days 1–7 (for Study 1) and over Days 1–5 (for Study 2). SOWS-G scores were analyzed using a Mixed-Effect Model Repeated Measure (MMRM) model of observed data (i.e. missing data were not imputed) for both Study 1 and Study 2. Data transformation was not required because normality was confirmed. The analyses included all randomized and treated subjects who had completed at least 1 post-dose SOWS-G.

The definition of study completion was the same in the label analysis as in the Studies 1 and 2 per-protocol analyses. The statistical analysis of study completion rate used in the label was a logistic regression model including fixed effects for treatment group and sex for Study 1 and a Fisher’s Exact test for Study 2. Completion rates were calculated on the population randomized and treated for both studies.

## 3. Results

### 3.1. Disposition and demographics

In Study 1, 603 subjects were randomized and 602 subjects received study drug: 222 received lofexidine 2.88 mg/day, 229 received lofexidine 2.16 mg/day, and 151 received placebo (randomized 3:3:2); in Study 2, 264 subjects were randomized and 263 subjects received study drug: 134 received lofexidine 2.88 mg/day and 129 received placebo (randomized 1:1).

In both studies, enrolled patients were predominantly white and male; heroin was the primary opioid used (Table 1). Study 1 had a lower proportion of Hispanic participants and a higher proportion of subjects using heroin as their primary opioid compared with Study 2 but in general the study populations were similar. Because race and ethnicity were collected as one variable in Study 2, white and black racial categories include non-Hispanics only. The Hispanic category includes both white and black Hispanics. Approximately, 60% of subjects in both studies provided positive urine screens for benzodiazepines, stimulants (cocaine and amphetamines) or cannabis. Duration of drug misuse was collected for Study 1 only with a mean duration of 8 or 9 years for each treatment group.

**Table 1.** Study background characteristics.

Characteristic	Study 1 *N* = 602			Study 2 *N* = 264	
	LFX 2.16 mg/day (*n* = 229)	LFX 2.88 mg/day (*n* = 222)	Placebo (*n* = 151)	LFX 2.88 mg/day (*n* = 134)	Placebo (*n* = 130)
Mean age, years (range)	35 (19–74)	35 (19–68)	36 (19–63)	36 (18–62)	38 (18–60)
Sex, %					
Male	71	71	71	75	76
Female	29	29	29	25	24
Race^a^, %					
White	74	71	78	47	59
Black or African American	23	21	17	28	21
Other	3	8	5	–	–
Ethnicity, %					
Hispanic/Latino	15	13	15	25	21
Primary opioid, %					
Heroin	86	82	81	61	64
Oxycodone	4	8	6	19	23
Hydrocodone	4	4	7	17	12
Other	5	5	6	3	1
Other illicit drugs^b^, %	59	57	66	60	59
Cannabinoids	28	36	27	28	22
Methamphetamines	16	20	15	2	6
Cocaine	18	20	12	31	38
Benzodiazepines	11	15	13	18	18
Amphetamines	12	15	9	<1	2
Buprenorphine	<1	1	<1	0	0
Methadone	0	<1	<1	0	<1
Barbiturates	<1	0	0	2	4
OOWS-H^c^, mean baseline score	4.9	4.9	5.2	5.42	5.46
Duration of drug misuse, years	9.3	7.9	8.8	NC	NC

^a^ For Study 2, the case report forms collected race and ethnicity as a single characteristic. “White” is non-hispanic white and “black” is non-hispanic black.

^b^ Based on urine screen at baseline.

^c^ Objective Opiate Withdrawal Scale-Handelsman (OOWS-H) baseline means. OOWS-H measures physical signs of opioid withdrawal; score ranges from 0 to 13.

Abbreviation. NC, not collected.

### 3.2. Efficacy: label analysis

In Study 1, mean SOWS-G score over Days 1–7 was 6.1 (SE: 0.35) for lofexidine 2.88 mg/day; 6.5 (SE: 0.34) for lofexidine 2.16 mg/day; and 8.8 (SE: 0.47) for placebo (mean difference for 2.88 mg: –2.75, SE: 0.58, *p* < .0001; for 2.16 mg: –2.33, SE: 0.58, *p* < .0001).

In Study 2, mean SOWS-G score over Days 1–5 was 7.0 (SE: 0.44) for lofexidine 2.16 mg/day and 8.9 (SE: 0.48) for placebo (mean difference: –1.91, SE: 0.65, *p* = .0037).

**Figure 2.**
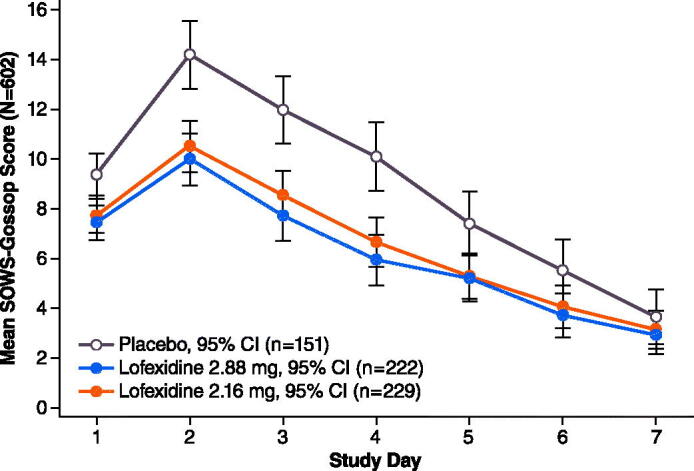
Study 1 mean^a^ SOWS-G Score on days 1–7. ^a^Least squares means from MMRM model; observed data only (no imputation of missing values). Abbreviations. CI, confidence interval; SOWS-G, Short Opioid Withdrawal Scale of Gossop. Population (*N* = 602) includes all subjects who received at least 1 dose of study medication and completed a post-dose SOWS-G.

**Figure 3.**
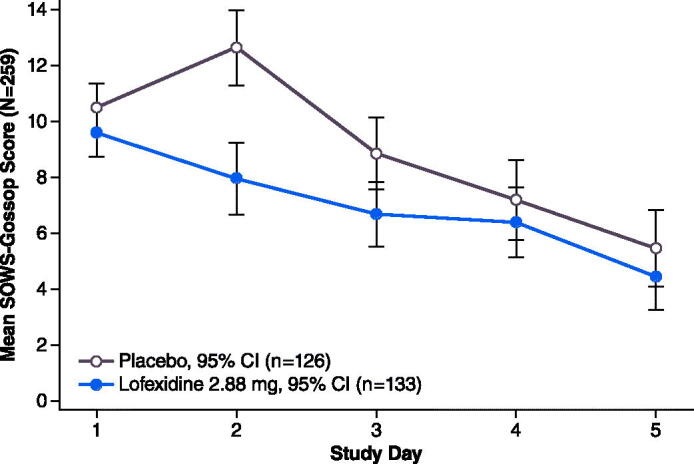
Study 2 mean^a^ SOWS-G Score on days 1–5. ^a^Least squares means from MMRM model; observed data only (no imputation of missing values). Abbreviations. CI, confidence interval; SOWS-G, Short Opioid Withdrawal Scale of Gossop. Population (*N* = 259) includes all subjects who received at least 1 dose of study medication and completed a post-dose SOWS-G.

Figures 2 and 3 depict mean SOWS-G scores by Study Day. Mean scores peaked on Day 1 or 2 then decreased steadily through the end of the treatment period.

A significantly greater proportion of lofexidine-treated subjects completed the trials compared with placebo-treated subjects. In Study 1, 28% of the placebo group, 41% of the lofexidine 2.16 mg group (*p* = .007) and 40% of the lofexidine 2.88 mg group (*p* = .02) completed 7 days of treatment. In Study 2, 33% of the placebo group and 49% of the lofexidine 2.88 mg group (*p* = .009) completed 5 days of treatment.

**Figure 4.**
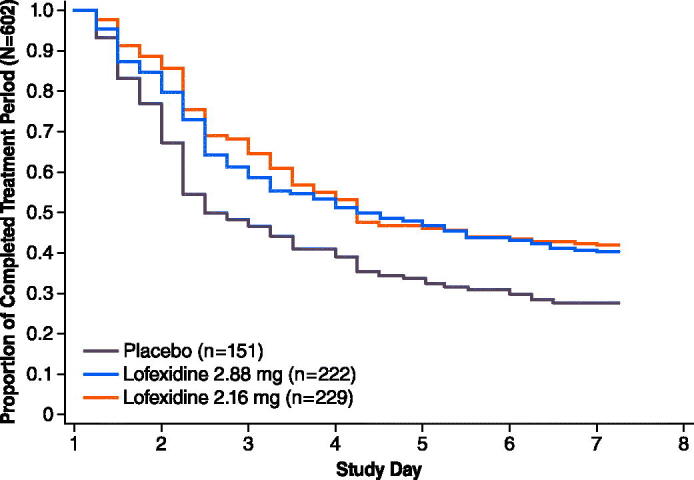
Study 1 subject retention over days 1–7. Population (*N* = 602) includes all subjects who received at least 1 dose of study medication.

**Figure 5.**
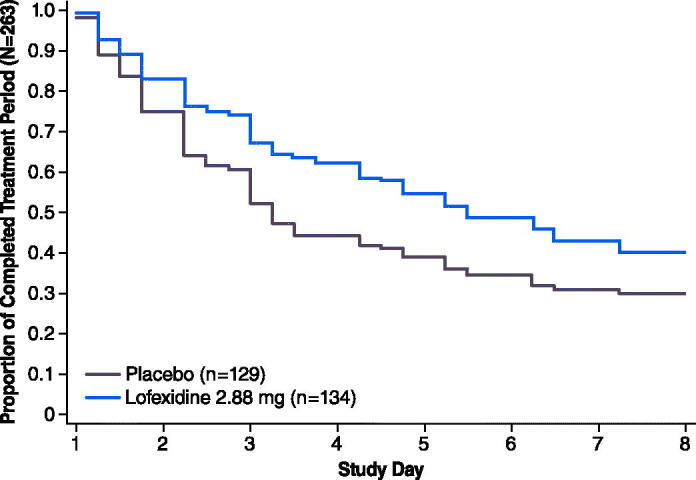
Study 2 subject retention over days 1–8. Population (*N* = 263) includes all subjects who received at least 1 dose of study medication.

In both studies substantially more placebo subjects discontinued by Days 2 and 3 compared with lofexidine subjects. The majority of discontinuations occurred by study Day 3 in both studies (Figures 4 and 5). Median time to last day of treatment was approximately 2 days longer for lofexidine treatment compared with placebo treatment during the double-blind periods. More placebo-treated subjects withdrew due to lack of efficacy compared with lofexidine-treated subjects.

The proportion of subjects using supportive medications (acetaminophen, antacids, bismuth, and zolpidem) in Study 1 was higher in the placebo group than in the lofexidine groups during the days of peak OWS (Days 2–5)^14^. These differences in proportion were not tested statistically and this analysis was not performed for Study 2.

### 3.3. Adverse events

Most AEs were mild or moderate in severity. AEs reported by more than or equal to 10% of subjects and more common with lofexidine treatment compared with placebo treatment are listed in Table 2. The most common adverse reactions (incidence ≥10% and notably more frequent than placebo) were orthostatic hypotension, bradycardia, hypotension, dizziness, somnolence, sedation, and dry mouth.

**Table 2.** Adverse events reported for ≥10% of lofexidine subjects and more frequently than placebo.

Study 1 *N* = 602				Study 2 *N* = 264		
Event, %	LFX 2.16 mg^a^ (*n* = 229)	LFX 2.88 mg^a^ (*n* = 222)	Placebo (*n* = 151)	Event, %	LFX 2.88 mg^a^ (*n* = 134)	Placebo (*n* = 130)
Insomnia	51	55	48	Insomnia	44	42
Orthostatic hypotension	29	42	5	–	–	–
Bradycardia	24	32	5	Bradycardia	10	2
Hypotension	30	30	1	Hypotension	25	1
Dizziness	19	23	3	Dizziness	22	7
Somnolence	11	13	5	–	–	–
Sedation	13	12	5	–	–	–
Dry mouth	10	11	0	Dry mouth	14	2
–	–	–	–	Anxiety	26	23
–	–	–	–	Fatigue	10	9

^a^ Assigned dose; mean average daily dose received was less than assigned dose due to dose-holds for out-of-range vital signs.

Abbreviation. LFX, lofexidine.

## 4. Discussion

As we deal with the impact of the opioid crisis in this country, lofexidine provides an important addition to pharmacotherapy options and is the only non-narcotic approved for the initial opioid discontinuation period, more commonly considered “detox.”

Lofexidine treatment compared with placebo reduced opioid withdrawal symptom severity as assessed by the SOWS-G. Lofexidine-treated subjects were significantly more likely to complete the double-blind treatment period and fewer lofexidine-treated subjects discontinued because of lack of efficacy compared with placebo-treated subjects. Although study completion rates were <50% (40–49%) in lofexidine groups, the increase in completion rate over placebo has important downstream effects on public health in the United States. Even a 10% reduction in opioid-dependence has the potential to reduce rates of opioid-associated death by several thousand persons per year and to decrease the annual economic burden from opioid use disorder by billions of dollars^15^^,^^16^.

The most common AEs that were notably greater than placebo were hypotension, orthostatic hypotension, dizziness, bradycardia, somnolence, sedation, and dry mouth; these events are consistent with the alpha_2_ central adrenergic receptor agonist mechanism of action. Package labeling recommends monitoring vital signs and symptoms related to orthostasis and bradycardia in inpatients, and ensuring outpatients are capable of self-monitoring these symptoms. An adjustment in dosing should be made in response to symptoms.

The choice of statistical methods for any data analysis requires unverifiable assumptions on the cause of missing data. The per-protocol specified analyses utilized a conservative approach to handle missing SOWS-G data, i.e. a “missing not at random” approach that imputed values for missing data. The label analyses were less conservative and used a “missing at random” approach with no imputation of missing data. Nonetheless, SOWS-G efficacy results presented in the label analyses, using a “missing at random” approach, were very similar to the per-protocol “missing not at random” analyses previously reported^9^^,^^10^. This confirmation of SOWS-G results across two different statistical analyses corroborates the efficacy of lofexidine for treatment of OWS. Additionally, the use of non-transformed data in the label analyses is easier to interpret and standardization of the SOWS-G endpoints (means over Days 1–7 and 1–5) allows comparison across studies.

Despite the small differences in study designs and patient populations (due to the passage of time between the development and enrollment into Studies 1 and 2) results were robust and consistent across studies, confirming the efficacy of lofexidine for mitigation of opioid withdrawal symptoms. Interestingly, although patient populations were similar in both studies for most background characteristics, heroin use was higher in Study 1. Because Study 2 completed in 2007 versus 2014 for Study 1, this likely reflects the increased use of heroin that occurred during that timeframe^17^.

The limitations of this analysis include lack of generalizability to populations other than adults dependent on short-acting opioids treated in inpatient settings. Studies are needed in more diverse patient types including persons with iatrogenic physical dependence, patients wishing to discontinue long-acting opioid agonists such as buprenorphine and methadone, and extended lofexidine treatment beyond 7–14 days, including outpatient treatment settings. Study strengths include the large study populations and quantity of safety data, the substantial proportions of females and minorities enrolled, and the inclusion of subjects with recent use of other illicit drugs (reflection of real-world use).

Lofexidine is the only non-opioid medication approved by FDA for the treatment of opioid withdrawal symptoms. It was approved in 2018 based on data from the 2 multicenter trials presented here, in addition to 3 other clinical trials and 16 clinical pharmacology studies. Clonidine is another alpha_2_-adrenergic receptor agonist that is commonly used off-label to treat opioid withdrawal symptoms. A 2016 Cochrane review of alpha_2_-adrenergic receptor agonists used to treat OWS reported that while both lofexidine and clonidine achieved similar efficacy, lower and better-tolerated doses of lofexidine could be used^18^. This may be due to pharmacologic differences between lofexidine and clonidine. Lofexidine but not clonidine has moderate agonist activity stimulating the 5HT1A receptor at relevant concentrations^19^. 5HT1A activation has been hypothesized to play a role in reducing OWS severity based on animal data^19^, but this potential mechanism of action needs to be confirmed by further research.

Opioid-dependent individuals often continue using opioids to avoid the severely uncomfortable symptoms that occur if they stop^5^^,^^6^. Lofexidine may be helpful to ease patient discomfort during early withdrawal, as demonstrated in the data presented here. Although lofexidine is not a treatment for opioid use disorder, these data provide evidence of its utility as a first-line intervention. Keeping a patient comfortable during opioid discontinuation allows the clinician to plan for long-term treatment with non-agonist or agonist therapy. This can be accomplished by any physician in an outpatient or inpatient setting. In the context of opioid use disorder, lofexidine should be used in conjunction with a comprehensive management program including psychosocial treatment and longer-term agonist or antagonist treatment. Patients who complete opioid discontinuation are at an increased risk of fatal overdose should they resume opioid use and patients and caregivers should be informed of increased risk of overdose due to reduced tolerance.

Once a physiologically dependent individual makes the important decision to discontinue opioids, successful management of OWS, especially during peak intensity (days 1–5), is a critical first step in an often difficult process of opioid cessation.

## Data Availability

Summary data tables can be requested from the corresponding author.

## Transparency

### Declaration of financial/other relationships

D.A. is a consultant for US WorldMeds, LLC, and for Alkermes, and reported receiving research support from Allergan, Otsuka, Janssen, and Alkermes. C.T. reported serving on speaker panels for Alkermes plc. M.P. and T.C. are employees of US WorldMeds, LLC. JDA peer reviewers on this manuscript have no relevant financial or other relationships to disclose.
